# Galectin-1 Promotes Vasculogenic Mimicry in Gastric Cancer by Upregulating EMT Signaling

**DOI:** 10.7150/jca.33765

**Published:** 2019-10-17

**Authors:** Xiaolan You, Qinghong Liu, Jian Wu, Yuanjie Wang, Jiawen Dai, Dehu Chen, Yan Zhou, Yanjun Lian

**Affiliations:** Department of Gastrointestinal Surgery, Hospital Affiliated 5 to Nantong University (Taizhou People's Hospital), Taizhou, Jiangsu province, China

**Keywords:** Galectin-1, Vasculogenic mimicry, Gastric cancer, Epithelial-to-mesenchymal transition

## Abstract

**Background**: Galectin-1 (Gal-1) expression was positively associated with vasculogenic mimicry (VM) in primary gastric cancer (GC) tissue, and that both Gal-1 expression and VM in GC tissue are indicators of poor prognosis. However, whether Gal-1 promotes VM, and by what mechanismsremains unknown.

**Methods**: To investigate the underlying mechanisms,wound healing assay, proliferation assay, invasion assay, and three-dimensional culture were used to evaluate the invasion, metastasis and promoted VM formation effects of the Gal-1. We monitored the expression level of sociated proteins in GC tissues, cell lines* in vitro* and nude mice tumorigenicity *in vivo* by immunohistochemistry and western blot.

**Results**: Gal-1 overexpression significantly promoted the proliferation, invasion, migration, and VM formation of MGC-803 cells. Gal-1 was associated with E-cadherin and vimentin *in vitro* and in clinical samples. The epithelial-to-mesenchymal transition (EMT) induced in MGC-803 cells by TGF-β1 was accompanied by Gal-1 activation and promotion of VM formation, while knockdown of Gal-1 reduced the response to TGF-β1, suggesting that Gal-1 promotes VM formation by activating EMT signaling. Overexpression of Gal-1 accelerated subcutaneous xenograft growth and facilitated pulmonary metastasis in athymic mice, enhanced the expression of EMT markers, and promoted VM formation* in vivo*.

**Conclusion**: Our results indicated that Gal-1 promotes VM in GC by upregulating EMT signaling; thus, Gal-1 and this pathway are potential novel targets to treat VM in GC.

## Introduction

Gastric cancer (GC) is one of the leading causes of death worldwide, with a high incidence and a high rate of lethal malignancies in China 1. In the past two decades, although great progress has been made in the diagnosis and therapy of GC, the overall survival rate remains unsatisfactory, especially in advanced disease 1, 2. Anti-angiogenesis treatment offers new hope for the treatment of GC, especially in chemotherapy-resistant patients 3. It was long believed that angiogenesis via the sprouting of endothelial vessels from existing ones was the exclusive form of tumor vascularization, and anti‑angiogenesis therapy was considered as a promising method to treat tumors 3, 4. However, with the wide application of angiogenesis inhibitors in clinical practice, it was established that the effect of antiangiogenic drugs was limited 5. This indicated that other modalities supply tumor nutrition in addition to endothelial vessels.

In 1999, Maniotis et al. 6 first reported an endothelial‑independent vascular channel, which formed with highly aggressive and metastatic melanoma cells, and this formation was termed vasculogenic mimicry (VM). Soon afterwards VM was identified in multivalent malignant tumors, especially in poorly differentiated malignancies, including gallbladder carcinoma 7, hepatocellular carcinoma 8, ovarian cancer 9, small cell lung cancer 10, glioblastoma 11, pancreatic duct carcinoma 12, and gastric adenocarcinoma 13. VM comprises a basement membrane, lined externally by tumor cells and a lack of endothelial cells in the internal lining 6, 14. Based on the endothelial cells visualized by CD34 immunohistochemical staining and the basement membrane visualized by Periodic acid‑Schiff (PAS) histochemical staining, this PAS-CD34 double staining showed that the endothelial‑dependent vasculature was CD34-PAS double positive, while VM was CD34 negative/PAS positive. Recent research indicated that VM might play an extremely important role in the biological behavior of human malignant tumors 15.

Galectin-1 (Gal-1) is a β-galactoside-binding protein that belongs to a 15-member protein family called the galectins 16. Among the 15 members of lectin family, Gal-1 appears to be the major player in cancer biology, which has stimulated significant research interest 16. Recent studies have found that extracellular Gal-1 is overexpressed in the stroma between tumor cells in multivalent malignant tumors 16-20. In addition, increased expression of Gal-1 correlated with a variety biological behaviors of malignant tumor, including cellular aggregation, cellular apoptosis, metastatic spread of cancer, tumor immunity, and angiogenesis 16-23. However, little is known about the association between Gal-1 and VM in GC.

Previously, we reported that Gal-1 expression was positively associated with VM in primary GC tissue, and both Gal-1 expression and VM in primary GC tissue are indicators of poor prognosis after gastrectomy 24. However, whether Gal-1 promotes VM, and by what mechanisms, remains unknown. In this study, we explored the role of Gal-1 in the development of VM and its underlying mechanisms in GC.

## Materials and methods

### Tumor tissue samples

As previously reported, 127 patients with gastric adenocarcinoma were enrolled in our study, no preoperative neoadjuvant chemotherapy or radiotherapy had been administered. All patients underwent radical gastrectomy at the Department of Gastrointestinal Surgery, Taizhou People's Hospital, Jiangsu province. GC tissues for immunohistochemistry (IHC) and histochemical staining were formalin-fixed. This study was approved by the Clinical Research Ethics Committee of our institute (TZRY-EC-12-068).

### Cell culture and lentiviral transduction

The human gastric adenocarcinoma cell line MGC-803 was obtained from the Type Culture Collection of the Chinese Academy of Sciences (Shanghai, China). The cells were maintained with Roswell Park Memorial Institute medium (RPMI; Thermo Scientific Hyclone, Waltham,MA , USA) supplemented with 10% FBS (fetal bovine serum; Thermo Scientific HyClone,), 100 U/ml penicillin, and 100 mg/ml streptomycin (GIBCO, Grand Island, Waltham, MA, USA), under conditions of 37 °C in a humidified atmosphere containing 5% CO_2_.

Lentiviral vectors carrying green fluorescent protein (GFP) and a puromycin resistance gene for Gal-1 overexpression and knockdown were commercially constructed by Genechem Co. Ltd. (Shanghai, China). MGC-803 cells were seeded on 6-well plates at 5 × 10^4^ cells per well before lentiviral transduction. Cells were transduced with the appropriate lentiviral vector containing at a multiplicity of infection (MOI) of 10 in the presence of 10 μg/ml polybrene (Sigma-Aldrich, Temecula, CA, USA). The appropriate fresh medium replaced the medium after incubation at 37 °C for 12 h. Puromycin (Sigma-Aldrich) was added to select for stably transduced cells at concentration of 2 µg/ml after incubation for 48 h. Stable transductants were cultured with puromycin at 0.5 μg/ml. Transduction efficiencies were evaluated 72 h after transduction by counting GFP positive cells under a fluorescent microscope (OLYMPUS-U-HGLGPS-IX73); positive transduction was further confirmed by quantitative real-time reverse transcription PCR (qRT-PCR) and western blotting.

### RNA extraction and real-time PCR

Total RNA was extracted using an RNeasy Mini Kit (Invitrogen, Waltham, MA, USA). First strand cDNA synthesis was performed using a reverse transcription kit (Takara, Shiga, Japan) and the real-time PCR analyses were conducted on an iQ5 Multicolor Real-Time PCR Detection System (Bio-Rad, Hercules, CA, USA) using the SYBR Green dye (Roche Diagnostics, Mannheim, Germany). The *GAPDH* gene was used as an internal control, and the data are shown as the fold change. The experiment was performed in triplicate. Primers for Gal-1, Vimentin (*VIM*), E-cadherin (*CDH1*), and *GAPDH* are shown in Table 1.

### Western blotting analysis

Total cell extracts and nuclear extracts were prepared using an extraction kit (Beyotime, Shanghai, China). 12% sodium dodecyl sulfate-polyacrylamide gel electrophoresis (SDS-PAGE) gels were used to separate 20 μg of cell lysates and the separated proteins were transferred to a nitrocellulose membrane (GE Healthcare Life Sciences, Pittsburgh, PA, USA). Blots were probed with rabbit monoclonal antibody against Gal-1 (Cell Signaling Technology, Danvers, MA, USA), rabbit monoclonal antibody against vimentin (Cell Signaling Technology), rabbit monoclonal antibody against anti-E-cadherin (Cell Signaling Technology), or mouse monoclonal antibody against GAPDH (Kang Cheng, Shanghai, China) antibodies at a dilution of 1:2000. Horseradish peroxidase (HRP)-conjugated goat anti-mouse immunoglobulin and HRP-conjugated goat anti-rabbit immunoglobulin were used as a secondary antibody at a dilution of 1:2000. The West Pico chemiluminescent Substrate (Pierce, Carlsbad, CA, USA) was used to visualize the immunoreactive protein bands, and densitometric image analysis software (Image Master VDS; Pharmacia Biotech) was used to quantify the visualized protein bands. The level of GAPDH was used as an internal reference. All experiments were performed in triplicate.

### Proliferation assay

The Cell Counting Kit-8 (CCK-8) assay was used as aqualitative index of cell proliferation.We plated ten thousandcells in 96-well microplates; CCK-8 (Dojindo, Kumamoto, Japan) was used according to the manufacturer's protocol. Briefly, we added 10μL of CCK-8 solution to each well, then incubated the samples for 1 h, and measured the absorbance at 450 nm. All experiments were performedin triplicate.

### Cell invasion assay

24-well Transwell units with polycarbonate filters (pore size, 8.0 μm; Corning, New York, USA) were used to measure the invasive ability of GC cells. In brief, the upper Transwell inserts were first coated with 100 µl Matrigel basement membrane (BD Biosciences, San Diego, CA, USA), and then 1× 10^5^ cells in 100 µL of serum-free RPMI medium were seeded on it. Medium (600 μl) containing 10% FBS was placed in the lower chamber as a chemoattractant. Cells were allowed to migrate at 37 °C for 24 h; non-invasive cells were removed with a cotton swab. 4% paraformaldehyde was used to fix the filters, and the cells were stained with a 0.05% crystal violet solution, and counted under a microscope with six randomly-selected fields at 100× magnification for each sample. Invasion assays were performed in triplicate.

### Wound-healing assay

When cells reach 80-90 % confluence in a 6-well plate, a sterile plastic pipette tip was scored across the cell surface to create a wound. The plates were washed three times to remove cellular debris using phosphate-buffered saline (PBS), and then incubated at 37 °C with serum-free medium containing 10μg/mL mitomycin C (to block proliferation). The wound was photographed at 0 h and 48 h. All experiments were performed in triplicate.

### Three-dimensional culture

A 24-well plate was coated with 200 μl growth factor-reduced Matrigel (BD Biosciences, San Diego, CA, USA), which was allowed to polymerize at 37 °C for 1 h, after which 1 × 10^5^ cells suspend in 600 μl of medium containing 10% FBS were plated on the surface of the gel and incubated at 37 °C for 24 h; three wells were provided for each group. Cells were then photographed under an inverted microscope (OLYMPUS-U-HGLGPS-IX73).

### Histological examination and IHC evaluation

IHC was performed according to our previous report 24. Slides were incubated with primary antibodies against Gal-1 (1:200), E-cadherin (1:200), or vimentin (1:200); the other steps and staining scores of Gal-1 were the same as in our previous report^24^. All specimens were stained in three sections using each antibody. E-cadherin and vimentin staining was defined as positive or negative. At a magnification of 400×, ten fields of each section were randomly selected under the microscope. Two independent pathologists blinded to the patient's clinical status assessed the results. The evaluation of staining result was accordance with the previous report^25^. The staining cells percentage was scored as 0 for 0-5%; 1 for 6-25%; 2 for 26-50% and 3 for 50-100%, the staining intensity was scores as 0 point for negative; 1 point for weak intensity; 2 points for moderate intensity and 3 points for strong intensity. The sum scores ≥3 points were considered as positive.

### CD34-PAS dual staining and VM evaluation

CD34-PAS dual staining and VM evaluation were performed according to our previous report 24. CD34-PAS dual staining of subcutaneous GC tumors and lung metastases was performed on formalin-fixed, paraffin-embedded tissue. Sections were cut at 4-μm thickness, and all specimens were stained in three sections using each antibody. At a magnification of 200×, ten fields of each section were randomly selected under the microscope to distinguish the positive or negative VM.

### Animal models

Subcutaneous GC implantation and lung metastasis models were established in five-week-old male athymic mice respectively. Mice were bought from the Comparative Medicine Centre of Yangzhou University (Yang Zhou, JiangSu, China), and the experiments were approved by the Ethic Committee of Yang Zhou University (YZU-EC-JS2352), breeded mice in the laminar flow cabinet under pathogen-free conditions. MGC-803 cells overexpressing Gal-1 (OE- Gal-1), with Gal-1 knock-down (KD- Gal-1), or wild-type control(WC) MGC-803 cells were separately inoculated into the right sides of the back or the tail vein of the athymic mice (2×10^6^ cells/mouse; n = 6/group). The diameters of the subcutaneous tumors were measured every three days. The mice in subcutaneous group were sacrificed on day 21 and the mice in lung metastasis group were sacrificed on day 50. The subcutaneous GC tumors and lung metastases were harvested and subjected to histological examination using hematoxylin and eosin (H&E) staining, IHC, or CD34-PAS dual staining.

### Statistical analysis

SPSS 16.0 (SPSS, Chicago, IL, USA) was used to conduct the statistical analysis. Continuous variables were expressed as the mean ± SE and Student's *t*-test was used to compare between groups. The Chi-squared test was applied to compare dichotomous variables. The nonparametric Spearman-Rho method was used to analyze the correlations. In all analyses, *P* < 0.05 was considered statistically significant.

## Results

### Gal-1 promotes invasiveness and VM-formation potentiality of GC cells

**Figure 1:**
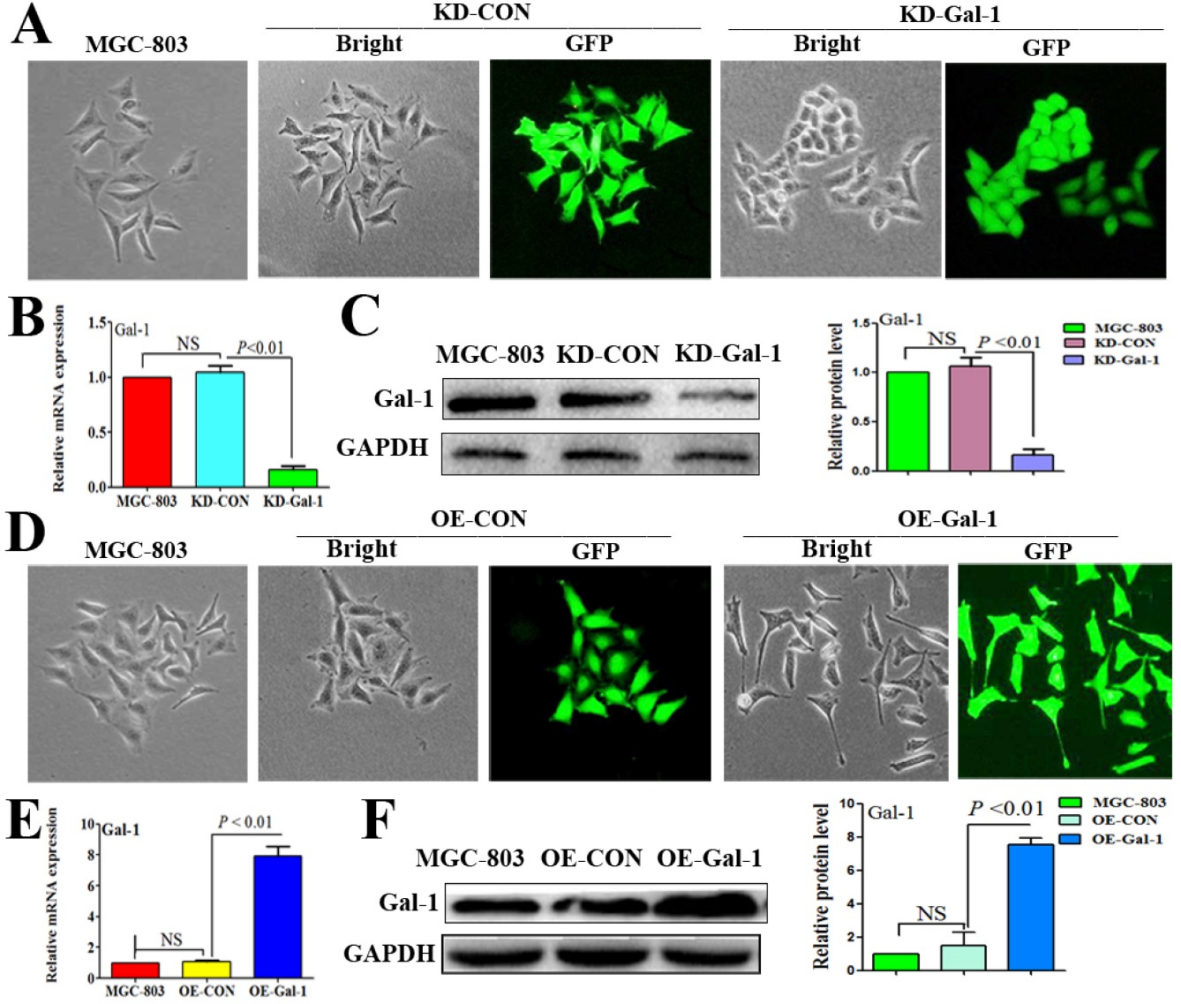
** Established MGC-803 cell lines with stable silencing of* Gal-1* (KD-Gal-1) and with overexpression of *Gal-1* gene (OE-Gal-1)** (A) Transfection of KD-Gal-1 lentiviruses induced morphological changes in MGC-803 cells, as confirmed by the GFP signal (magnification: ×200). (B and C) Stable knockdown of Gal-1 expression in MGC-803 cells was assessed using qRT-PCR and western blotting. (D) MGC-803 cells overexpressing Gal-1 (OE-Gal-1) exhibited a more elongated architecture than MGC-803 infected with the overexpression control virus (OE-CON), (magnification: ×200). (E and F) Stable overexpression of Gal-1 in MGC-803 cell line was assessed using qRT-PCR and western blotting. The bars represent the mean of three independent experiments ± SD.

**Figure 2:**
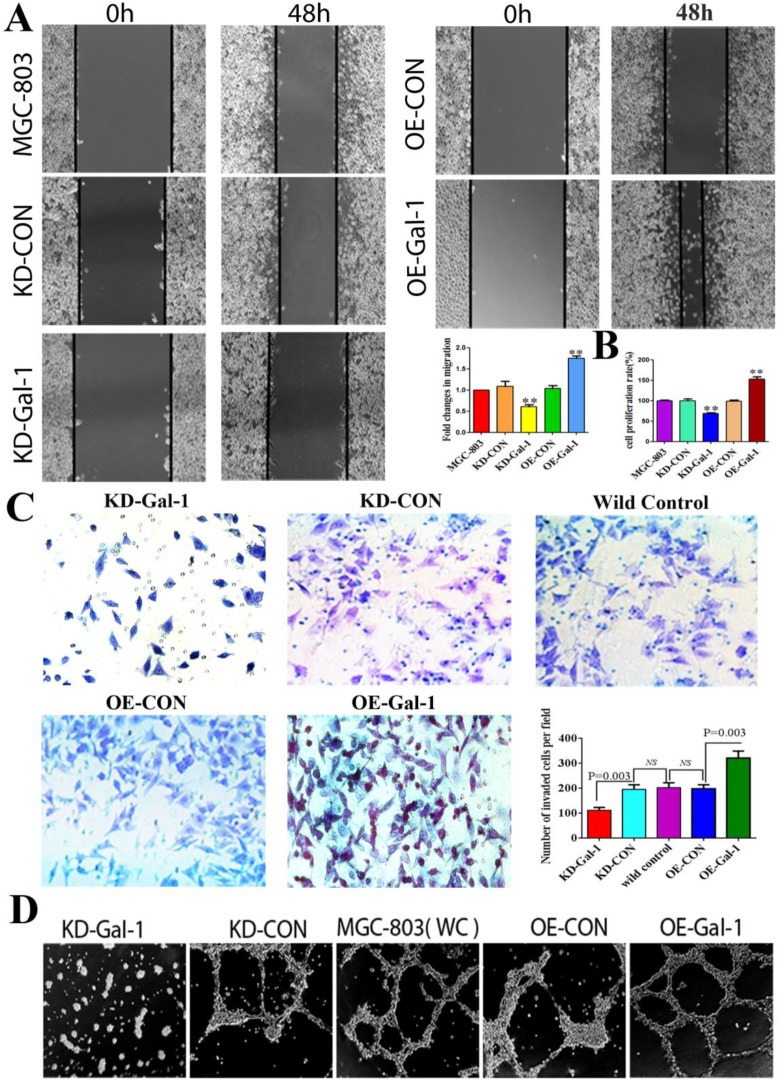
** Gal-1 promotes proliferation, invasiveness and VM formation potential of gastric cancer cells**. (A) *Gal-1* overexpression in MGC-803 cells exhibited significantly enhanced migration capacity after 48 h in a wound assay, while knockdown of *Gal-1* in MGC-803 cells destroyed their ability to migrate. Magnification: ×40. * P < 0.05, ** P < 0.01. These results are presented as mean ±SD of three independent experiments. (B) Gal-1 promotes the proliferation of MGC-803 cells. (C) Matrigel invasion assay showing MGC-803 cells with Gal-1 overexpression or knockdown; the numbers of invaded cells were quantified in six randomly selected fields at ×200 magnification. Bars represent the mean number of invaded GC cells (six fields/sample). (D) Matrigel three-dimensional culture showing that *Gal-1* overexpression increased the capacity for tube-formation in MGC-803 cells, while knockdown of Gal-1 in MGC-803 cells destroyed their ability to form tube-like structures. Original magnification, 40x, n=3.

To investigate whether Gal-1 contributes to VM, two lentiviruses were constructed to establish MGC-803 cell lines with stable overexpression of the Gal-1 gene (OE-Gal-1) and Gal-1 silenced (KD-Gal-1), which were confirmed by detecting the GFP signal (Figure 1A and 1D), qRT -PCR (Figure 1B and 1E), and western blotting (Figure 1C and 1F). Compared with MGC-803 cells infected with the knockdown control virus (KD-CON), knockdown of Gal-1 resulted in MGC-803 adopting a fusiform shape (Figure 1A). OE-Gal-1 cells exhibited more elongated architecture than MGC-803 infected with the overexpression control virus (OE-CON) (Figure 1D). As shown in Figure 2A, OE-Gal-1 cells exhibited a significantly enhanced migration capacity compared with cells infected with the OE-CON lentiviruses and the wild-type control, while KD-Gal-1 cells lost their ability to migrate. By the method of Proliferation assay, we observed that the proliferation of MGC-803 cells was increased with elevated Gal-1 expression (Figure 2B). Gal-1 overexpression in MGC-803 increased cell invasiveness, while knockdown of Gal-1 in MGC-803 cells destroyed their ability to invade (Figure 2C). Interestingly, Gal-1 overexpression increased the cells' capacity for tube-formation in MGC-803 cells, while KD-Gal-1 cells lost their ability to form tube-like structures (Figure 2D). These results suggested that Gal-1 plays an important role in the promotion of invasion and the induction of VM formation in GC.

### Gal-1 expression was elevated in an EMT model

**Figure 3:**
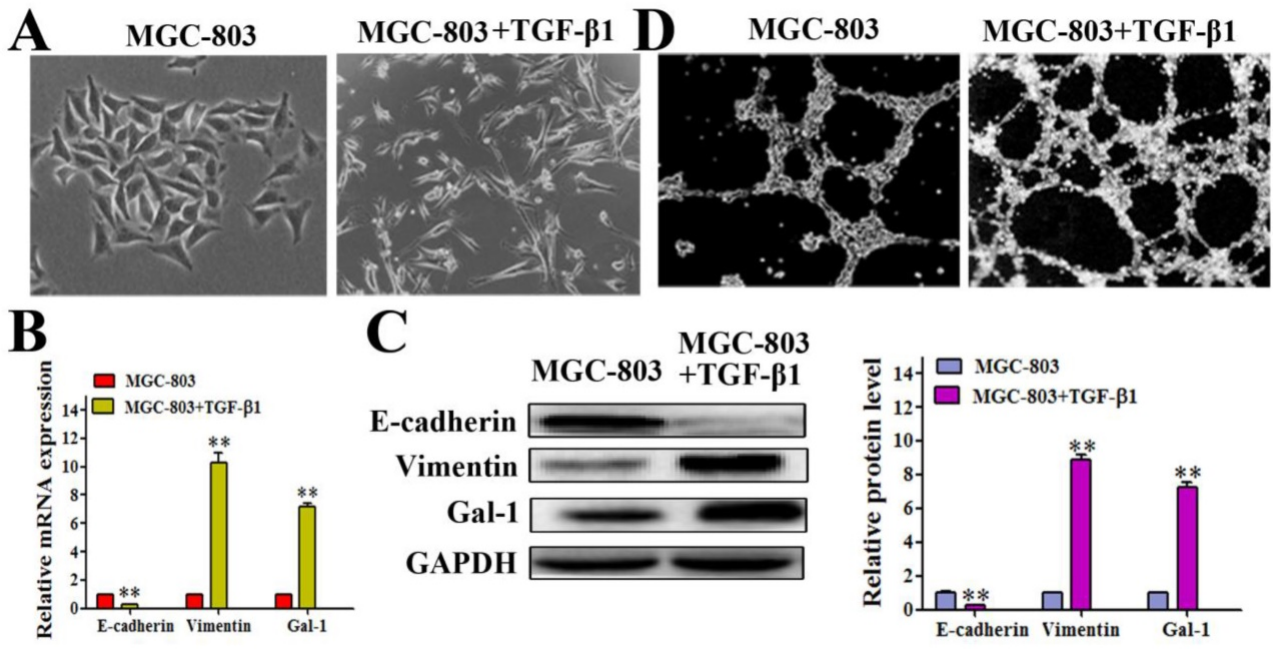
Gal-1 expression was elevated in a TGF-β1-induced EMT model (A) MGC-803 cells treated with 10 ng/ml TGF-β1 for 24 hours exhibited a more elongated architecture; original magnification, 200x (B and C) qRT-PCR and Western blot showing an increased Vimentin level, decreased E-cadherin level, and increased Gal-1 level. * *P* < 0.05, ** *P* < 0.01. The bars represent the mean of three independent experiments ± SD. (D) Typical tube-like structures increased in 3-dimensional culture in the TGF-β1-induced EMT model; original magnification, 40×. n=3.

It has been reported that the EMT contributes to tumor cell plasticity and is the key step during VM formation 26-28. As such, we hypothesized that Gal-1 contributes to VM formation by inducing EMT. We treated MGC-803 cells with 10 ng/ml transforming growth factor beta 1 (TGF-β1) for 24 hours, which caused the cells to exhibit a more elongated architecture than MGC-803 control cells (Figure 3A). In addition, the TGF-β1-treated cells showed substantially reduced levels of the epithelial marker E-cadherin, and increased the levels of the mesenchymal marker vimentin both in mRNA and protein level (Figure 3B and 3C). Interestingly, treating MGC-803 cells with TGF-β1 could significantly increase the expression of Gal-1 (Figure 3C), which suggested a possible role for Gal-1 in the EMT of GC cells. Meanwhile, typical tube-like structures increased in the 3-dimensional culture in the TGF-β1 induced EMT model (Figure 3D). This finding suggested a possible role of Gal-1 in inducing EMT and promoting VM formation in GC.

### Gal-1 induced VM in GC cells by upregulating EMT signaling

**Figure 4:**
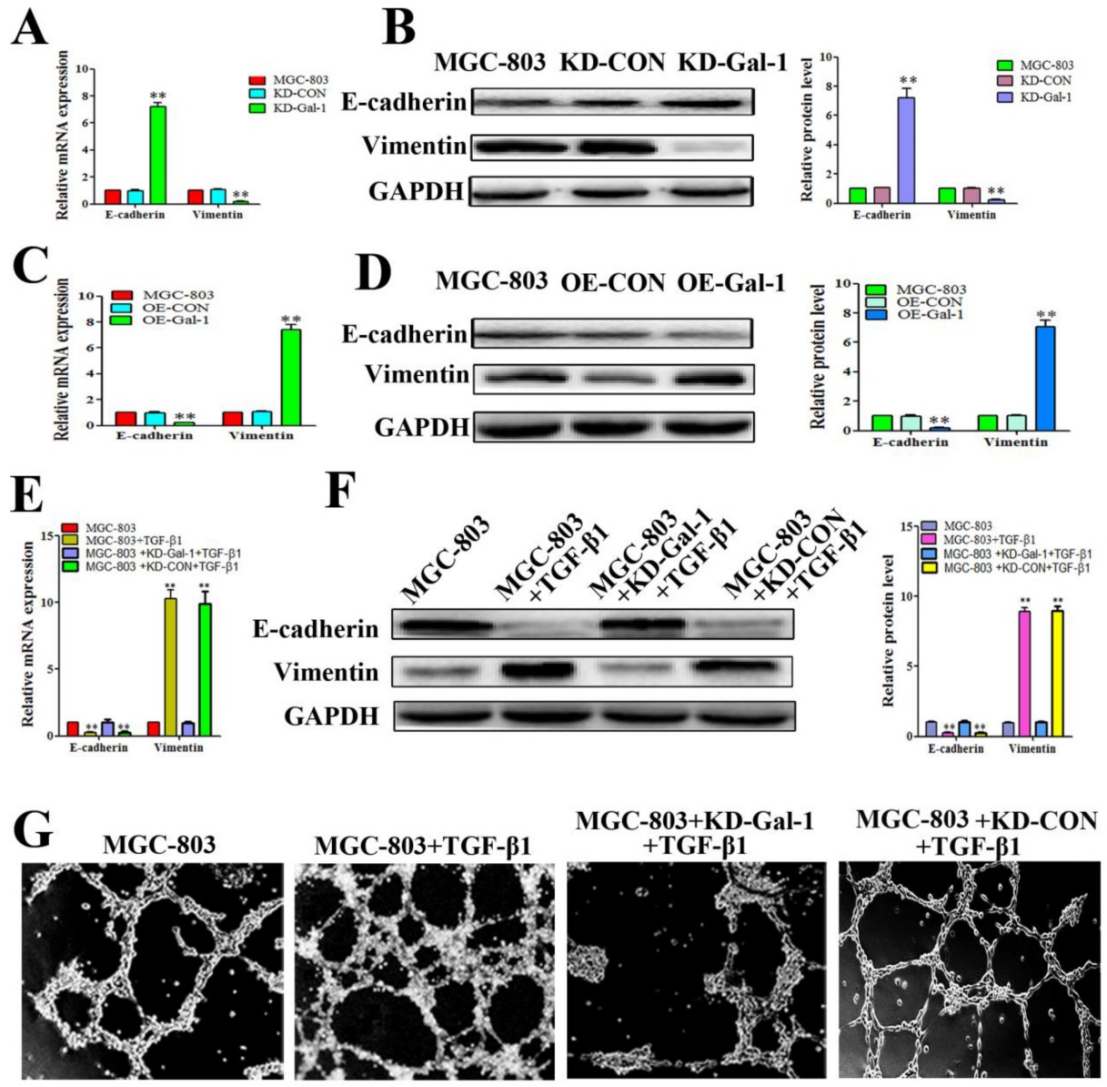
** Gal-1 overexpression led to EMT and form VM in gastric cancer cells** (A and B) MGC-803 cells with *Gal-1* knockdown (KD-Gal-1) exhibited significantly increased of E-cadherin and decreased vimentin. (C and D) Over expression of *Gal-1* (OE- Gal-1) in MGC-803 cells decreased E-cadherin expression and increased vimentin expression. (E and F) *Gal-1* knockdown caused MGC-803 cells to lose their responsiveness to TGF-β1 induction. * P < 0.05, ** *P* < 0.01. The bars represent the mean of three independent experiments ± SD. (G) Stimulation with TGF-β1 increased the ability of MGC-803 to form VM, while *Gal-1* knockdown MGC-803 cells failed to form VM. n=3.

Based on the finding that Gal-1 is increased in MGC-803 cells in the EMT model treated with TGF-β1, and that the TGF-β1-induced EMT model promoted typical tube-like structures in MGC-803 cells, we next determined whether overexpression of Gal-1 promoted the VM formation of GC cells by inducing EMT. MGC-803 cells with Gal-1 knockdown (KD-Gal-1) exhibited significantly increased E-cadherin and decreased vimentin levels (Figure 4A and 4B), while overexpression of Gal-1 (OE-Gal-1) in MGC-803 cells decreased the E-cadherin level and increased the vimentin level (Figure 4C and 4D). We then assessed the TGF-β1 treatment in MGC-803 cells and its counterpart which with Gal-1 knockdown and treat with TGF-β1 in MGC-803 cells, including the EMT marker proteins and VM formation ability. Similar to previous studies, treatment of MGC-803 cells with TGF-β1 for 24 hours increase the level of vimentin and decreased that of E-cadherin (Figure 4E and 4F). However, Gal-1 knockdown cells lost their responsiveness to TGF-β1 induction (Figure 4E and 4F). Meanwhile, stimulation with TGF-β1 increased the ability of MGC-803 to form VM, while the ability of VM formation was decreased in the Gal-1 knockdown cells (Figure 4G). These results suggested that Gal-1 induced VM-formation in GC cells by upregulating EMT signaling.

### Gal-1 associates with EMT-related biomarkers in GC tissues

**Figure 5:**
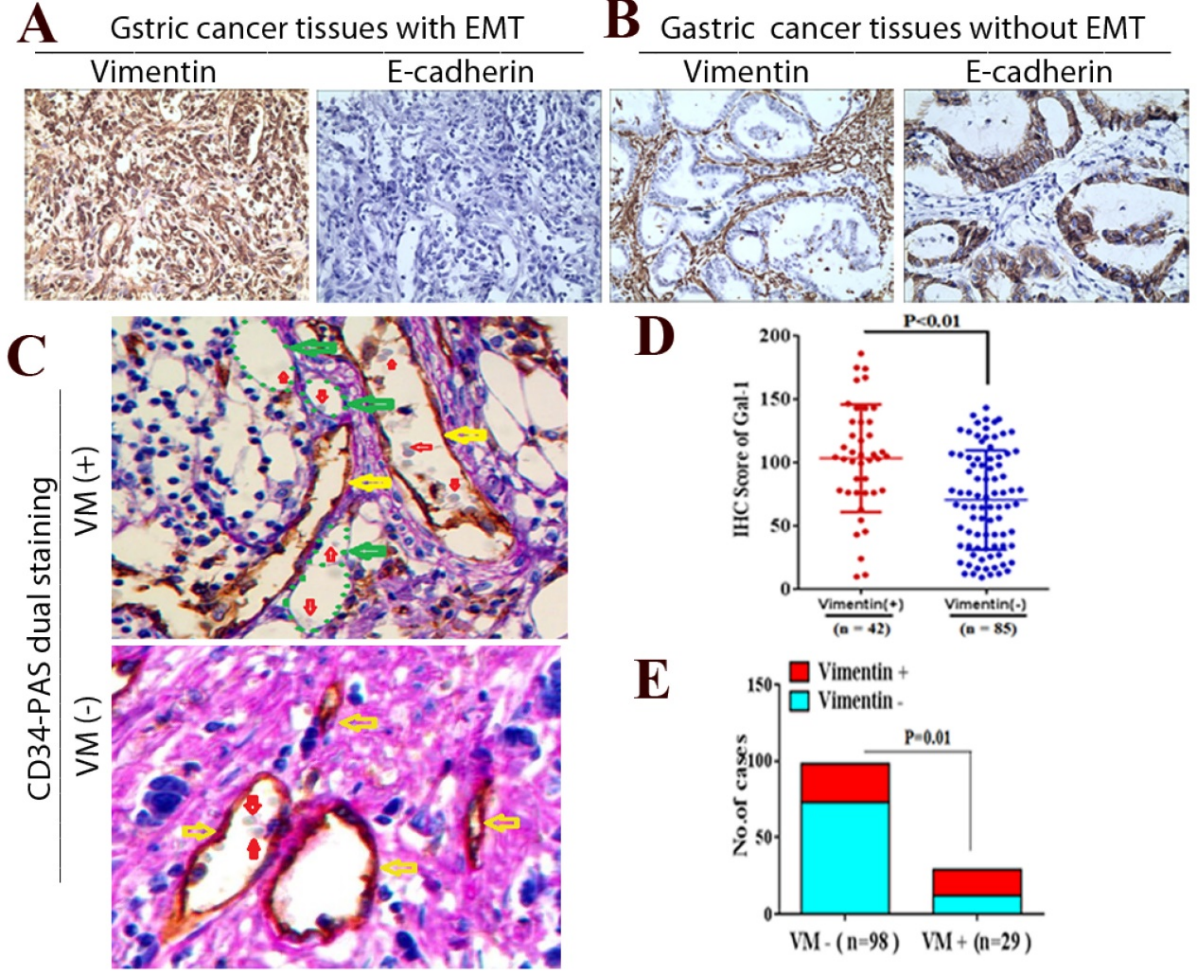
** Gal-1 associates with EMT-related biomarkers in GC tissue.** (A and B) Vimentin and E-cadherin are identified with IHC in gastric cancer tissues with EMT and without EMT. (C) CD34-PAS staining showing endogenous cell-dependent vessels (yellow arrows) ,VM (green dotted line) and red blood cells(red arrow) in GC specimen with VM and without VM. Original magnification: ×400. (D) The Gal-1 IHC scores in primary tumors with EMT were significantly different to Gal-1 score in primary tumors without EMT (*P* < 0.01). (E) EMT status was compared between vimentin positive GC cases and vimentin negative cases (*P* = 0.01).

To further determine whether Gal-1 can promote EMT and induce VM in GC tissue, we examined EMT-related markers in GC tissue using IHC, and identified the VM using CD34/PAS double-staining. We identified positive vimentin staining in both the tumor stroma and GC cells (Vimentin +) in 42 cases, and E-cadherin was decreased in those samples (Figure 5A). In addition, vimentin was expressed only in mesenchymal tissues (Vimentin -) in the remaining 85 samples, and E-cadherin was expressed only in epithelial tissues (Figure 5B). Moreover, the IHC scores of Gal-1 in primary tumors in the 42 vimentin-positive cases were higher than those in 85 vimentin-negative cases (Figure 5D). There was a significant association between the IHC score of Gal-1 and the expression of vimentin (*P* <0.01). In the 127 cases, CD34-PAS double staining revealed VM in 29 cases (22.8%); the remaining 98 cases were VM-negative (77.2%) (Figure 5C). Furthermore, 17/29 VM-positive cases also showed vimentin overexpression, while only 25/98 VM-negative cases had vimentin overexpression (Figure 5E). There was a significant association between the expression of vimentin and VM (*P* = 0.01,). These results suggested that EMT is associated with VM formation in GC, and that Gal-1 contributes to this process.

### Gal-1 promotes VM in vivo

**Figure 6:**
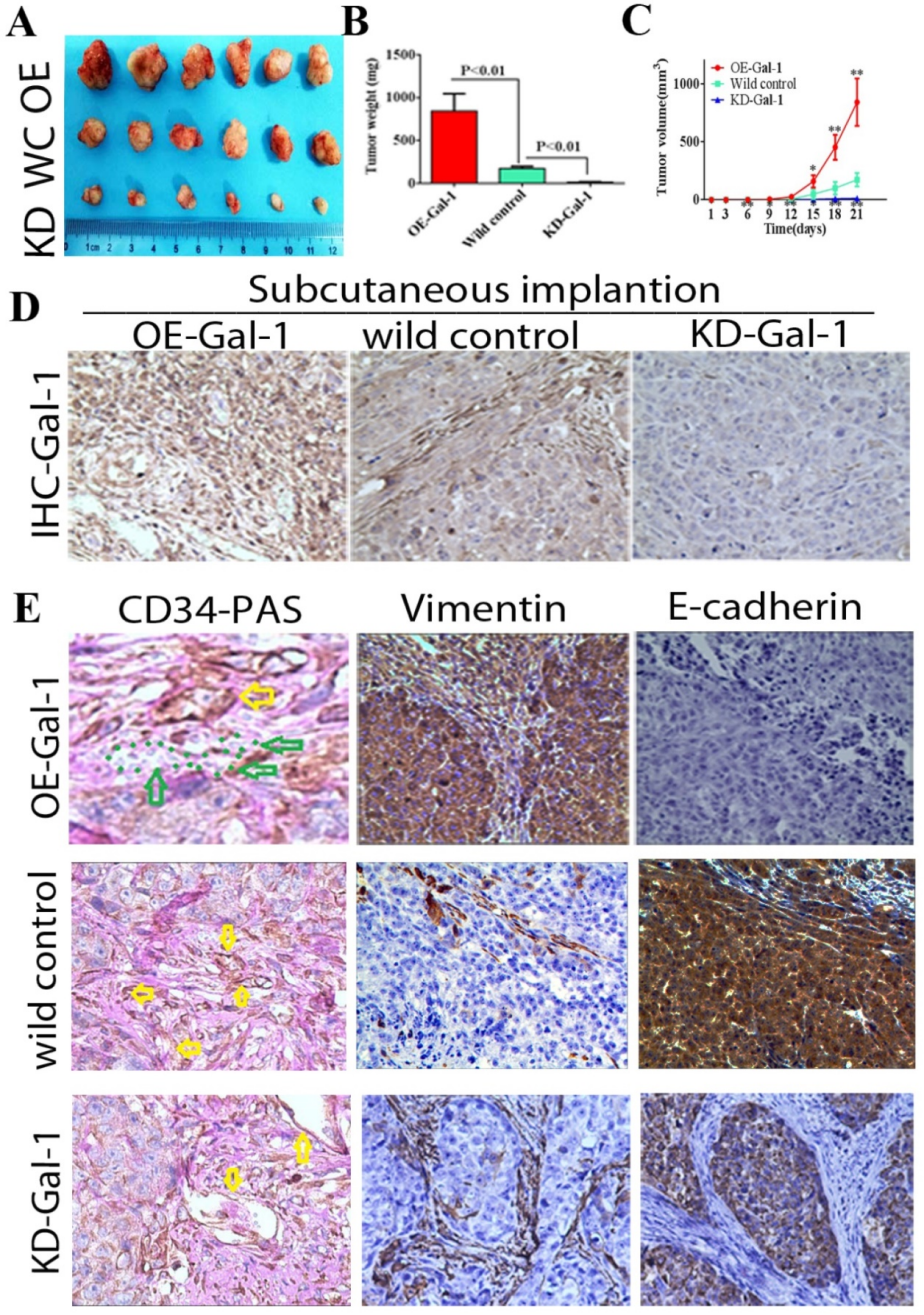
** Manipulation of Gal-1 expression influences gastric cancer xenograft growth and VM formation** (A) Gal-1 overexpression induced MGC-803 to form bigger subcutaneous xenograft Tumor weight (B) and volume (C) is expressed as the mean ± SE. * P <0.05, ** *P*<0.01, n = 6. (D) Representative Gal-1-IHC; Magnification: ×400. (E) CD34-PAS staining showing endogenous cell-dependent vessels (yellow arrows) and VM (green dotted line) in OE -Gal-1, wild control and KD-Gal-1 gastric cancer xenograft, and immunostaining shown vimentin and E-cadherin in those samples. Original magnification: ×400.

Through* in vitro* experiments and clinical analysis, we demonstrated that Gal-1 promotes VM formation in GC through an EMT-mediated process. Subcutaneous GC implantation and lung metastasis models in athymic BALB/c mice (*n* = 6/group) were generated to further determine the relationship between Gal-1 and GC growth, metastasis, VM formation, and the EMT-related markers. Twenty-one days after subcutaneous GC implantation, tumors in the Gal-1 overexpression group (OE) were larger and heavier than wild-type control group (WC), and the tumors in the Gal-1 knockdown groups (KD) were significantly smaller and lighter than those in the wild-type control group (Figure 6A, B; *P* < 0.01). From day 6 onward, the tumor volume in the KD-Gal-1 group was significantly lower compared with that in the wild-type control, and the volumes of the tumors in the OE-Gal-1 group were significantly higher after day 15 (*P* < 0.01 and *P* < 0.05; Figure 6C). Compared with the wild-type control group, Gal-1 expression was observed to be elevated in the OE-Gal-1 group using immunostaining, while KD-Gal-1 group was negative for Gal-1 expression (Figure 6D). CD34/PAS staining indicated that VM was significantly increased in the OE-Gal-1 group, and immunostaining showed positive vimentin staining in both the tumor stroma and tumor cells, while E-cadherin staining was negative in those samples. By contrast, VM was not observed in the tumor tissues in the KD-Gal-1 and wild control groups, and vimentin was detected only in mesenchymal tissues (Figure 6E).

**Figure 7:**
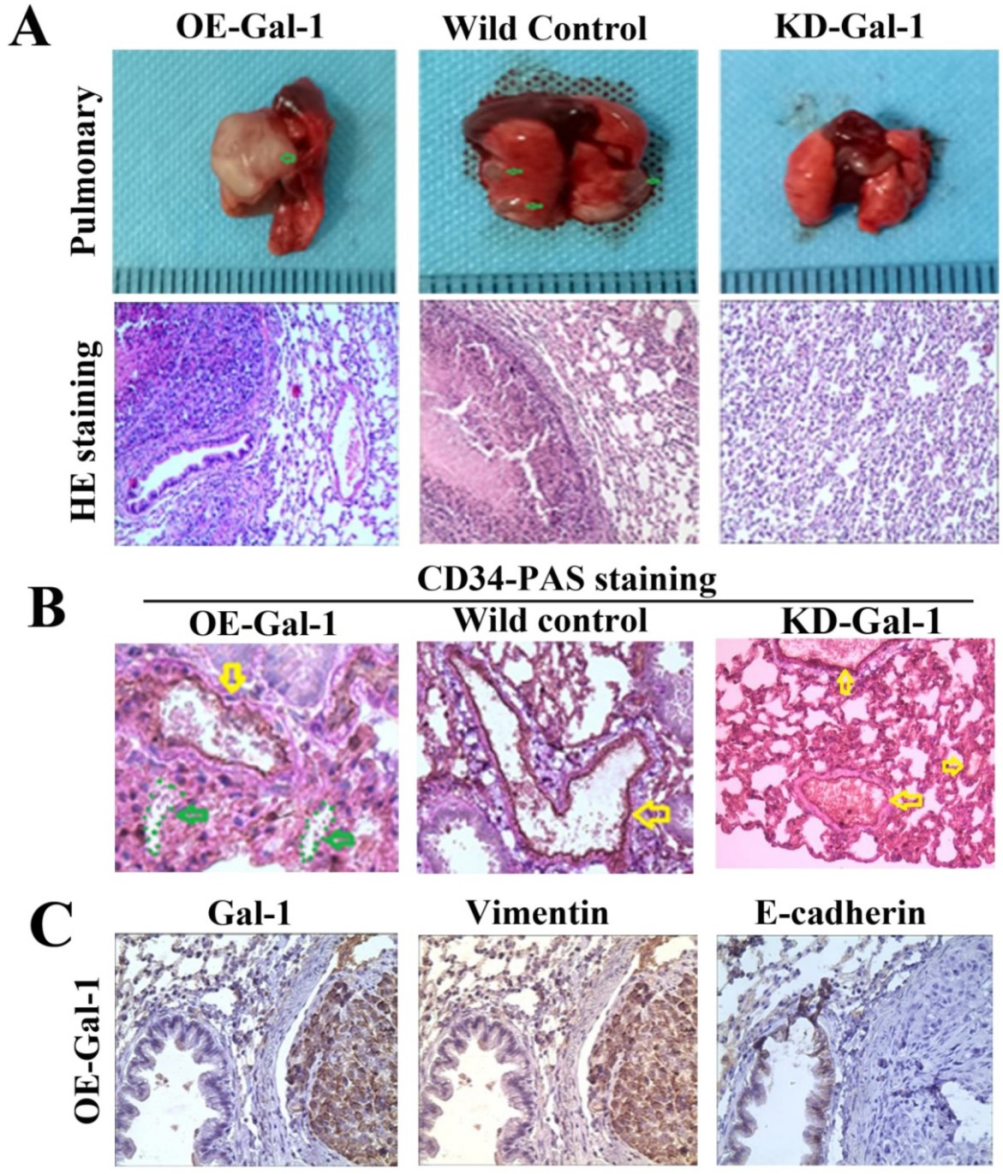
** Manipulation of Gal-1 expression influences MGC-803 GC cells pulmonary metastases and VM formation.** (A) Representative images of metastasis (green arrows) in the lungs at 50 days after inoculation, representative images of HE. Original magnification: ×200. Metastases were frequent in the (B) CD34-PAS staining showing endogenous cell-dependent vessels (yellow arrows) and VM (green dotted line) in pulmonary metastases. (C) Immunostaining shown Gal-1, vimentin and E-cadherin in pulmonary metastases. Magnification: ×400.

Fifty days after the generation of the lung metastasis models, larger and more extensive pulmonary metastases were found in every one in the OE-Gal-1 group, 4 cases had pulmonary metastasesin the wild-type control group, while the KD- Gal-1 group did not have any pulmonary metastases (*P* = 0.002; Figure 7A). H&E staining showed pulmonary metastases in the OE-Gal-1 group and the wild-type control group (Figure 7A). CD34-PAS double staining and immunostaining were used to examine the VM and EMT-related markers in the pulmonary metastases. CD34/PAS staining indicated that VM was significantly increased in the pulmonary metastases tissues in the OE-Gal-1 group (Figure 7B). Immunostaining showed that vimentin was positive in both the tumor and tumor stroma in the OE-Gal-1 group, and E-cadherin was negative in the tumor tissues (Figure 7C). These results suggested that Gal-1 plays an important role in GC invasion, metastasis, and VM-formation, and that EMT potentially contributes to this process.

## Discussion

VM formation in malignant tumor tissues tend towards tumor metastasis and have poor prognosis 29-33, which might be considered as an important target for anticancer therapy. Therefore, the study of the mechanism and the treatment of VM formation are required to improve treatments for cancer. In our previous study 24, we observed that VM formation was positively associated with the expression of Gal-1 in primary GC tissue. However, whether Gal-1 promotes VM and its mechanisms were unknown. The present study aimed to determine the role of Gal-1 in VM formation in GC.

To explore whether VM formation is associated with Gal-1 *in vitro*, we successfully constructed two lentiviruses for overexpression and knockdown of Gal-1, which were transduced into MGC-803 cells, separately. Overexpression of Gal-1 promoted the proliferation, invasion, migration, and VM formation of MGC-803 cells. Meanwhile knockdown Gal-1 expression in MGC-803 decreased the proliferation, invasion, migration, and VM formation of the cells. Previous studies indicated that Gal-1 promotes multiple metastatic processes via adhesion of tumor cells to the extracellular matrix, binding of cellular matrix glycoproteins, and enhancing proteolytic enzyme pathways 34, 35. In this study, we observed that Gal-1 enhanced the proliferation, invasion, migration, and VM formation ability of GC cells and promote the growth and metastasis of xenograft tumors in nude mice, which agreed with previous findings.

In the *in vivo* experiment, we observed that high expression of Gal-1 in MGC-803 cells not only promoted tumor growth, but also promoted pulmonary metastases in nude mice. Furthermore, we found that the xenograft tumors of overexpression group showed more VM formation. Previous studies reported that there was a junction between endothelial-lined blood vessels and VM formation in tumor tissues 36, 37. Through this junction, tumor cells lining the VM surface could directly participate in blood circulation, significantly increasing their transfer opportunities. This finding could explain the mechanism of Gal-1's promotion of metastasis in GC through the formation of VM.

Gal-1 can be detected in a variety of malignant tumors, and a high level of Gal-1 was linked to poor prognosis where Gal-1 was shown to induce EMT^16^-^22^, ^34^, ^35^, ^38^. In addition, many studies showed that EMT is an essential step in VM formation^26^-^28^. Therefore, we hypothesized that Gal-1 promotes the formation of VM in GC through EMT. To test this hypothesis, we established a classic EMT model for MGC-803 cells by treating them with TGF-β1. Interestingly, we found that the expression of Gal-1 was significantly increased in the EMT model, and that Gal-1 overexpression inhibited the expression of the epithelial marker E-cadherin and promoted the expression of the mesenchymal marker vimentin in MGC-803 cells. In clinical specimens, we found that the IHC score of Gal-1 was associated with EMT-related markers in GC tissue. Furthermore, we found Gal-1 formed VM not only in GC cells when cultured in Matrigel, but also in xenograft tumors and GC specimens. The *in vivo* and *in vitro* results suggested that Gal-1 expression is related to EMT markers expression and VM formation. Taken together, our results suggest that Gal-1 may promotes EMT and forms VM in GC. Furthermore, we found that a knockdown of Gal-1 suppressed the expression of EMT markers stimulated by TGF-β1. The results suggest that the Gal-1 / TGF-β1 axis may be involved in Gal-1-mediated EMT in GC and Gal-1 may promote the EMT in GC by enhancing TGF-β signaling, which is consistent with findings in a previous study^39^ .

Paz A, et al.^40^ found that Gal-1 promotes H-Ras activation intracellularly, and Ras signaling can enhance Sonic hedgehog (*SHH*) expression, leading to activation of Hedgehog (Hh) signaling. Glioma-associated oncogene -1 (Gli-1) is activated by Hh signaling to induce and promote EMT of GC 41. Therefore, -Gal-1 may alsopromotes EMT in GC via the Ras/Hh/Gli-1 signaling pathway.So the specific molecular mechanisms of Gal-1's promotion VM through EMT are needed further study.

Previous studies showed that Gal-1 and VM were identified in multivalent malignant tumors 7-13, 16-19. Gal-1 was also shown to induce EMT 16-22, 34, 35, 38, while EMT is an essential step in VM formation 26-28. However, whether Gal-1 also promotes the VM formation in other tumors remains to be investigated. Further experiment is planned to explore the hypothesis that Gal-1 promote EMT and VM in GC or other tumors.

## Conclusions

In conclusion, we implemented substantial experiments to explore the mechanism of Gal-1 induced VM in GC. We confirmed that Gal-1 promotes GC progression through VM formation by activating the EMT pathway. Our results will provide therapeutic targets for the development of new drugs to treat GC.

## Figures and Tables

**Table 1:** Primers used for qRT-PCR

Gene	Forward sequence	Reverse sequence
Gal-1 Ensembl:ENSG00000100097	GCTGAACCTGGGCAAAGACAG	GTTGAGGCGGTTGGGGAACTT
Vimentin Ensembl:ENSG00000026025	TGAATACCAAGACCTGCTCAA	ATCAACCAGAGGGAGTGA ATC
E-cadherin Ensembl:ENSG00000039068	TCGTCACCACAAATCCAGTG	CATTCACATCAAGCACATCC
GAPDH Ensembl:ENSG00000111640	TGACTTCAACAGCGACACCCA	CACCCTGTTGCTGTAGCCAAA

## Abbreviations

EMT: epithelial-to-mesenchymal transition
FBS: fetal bovine serum
Gal-1: galectin-1
GC: gastric cancer
GFP: green fluorescent protein
Gli-1: Glioma-associated oncogene-1
H&E: hematoxylin-eosin
Hh: Hedgehog
HRP: Horseradish peroxidase
IHC: immunohistochemistry
KD-Gal-1: Gal-1 knock-down
MOI: multiplicity of infection
OE-Gal-1: overexpressing Gal-1
PBS: phosphate-buffered saline
qRT-PCR: quantitative real-time PCR
PAS: Periodic acid‑Schiff
RPMI: Roswell Park Memorial Institute medium
SDS-PAGE: sodium dodecyl sulfate-polyacrylamide gel electrophoresis
SHH: Sonic hedgehog
VIM: Vimentin
VM: Vasculogenic mimicry
WC: wild-type control
